# Inter- and Intra-Individual Differences in EMG and MMG during Maximal, Bilateral, Dynamic Leg Extensions

**DOI:** 10.3390/sports7070175

**Published:** 2019-07-18

**Authors:** John Paul V. Anders, Cory M. Smith, Joshua L. Keller, Ethan C. Hill, Terry J. Housh, Richard J. Schmidt, Glen O. Johnson

**Affiliations:** 1 Department of Nutrition and Human Sciences, University of Nebraska-Lincoln, Lincoln, NE 68510, USA; 2 College of Health Sciences, Kinesiology, University of Texas at El Paso, El Paso, TX 79968, USA; 3 School of Kinesiology & Physical Therapy, Division of Kinesiology, University of Central Florida, Orlando, FL 32816, USA

**Keywords:** electromyography, mechanomyography, neuromuscular, fatigue, isokinetic, maximal, dynamic, vastus lateralis

## Abstract

The purpose of this study was to compare the composite, inter-individual, and intra-individual differences in the patterns of responses for electromyographic (EMG) and mechanomyographic (MMG) amplitude (AMP) and mean power frequency (MPF) during fatiguing, maximal, bilateral, and isokinetic leg extension muscle actions. Thirteen recreationally active men (age = 21.7 ± 2.6 years; body mass = 79.8 ± 11.5 kg; height = 174.2 ± 12.7 cm) performed maximal, bilateral leg extensions at 180°·s^−1^ until the torque values dropped to 50% of peak torque for two consecutive repetitions. The EMG and MMG signals from the vastus lateralis (VL) muscles of both limbs were recorded. Four 2(Leg) × 19(time) repeated measures ANOVAs were conducted to examine mean differences for EMG AMP, EMG MPF, MMG AMP, and MMG MPF between limbs, and polynomial regression analyses were performed to identify the patterns of neuromuscular responses. The results indicated no significant differences between limbs for EMG AMP (*p* = 0.44), EMG MPF (*p* = 0.33), MMG AMP (*p* = 0.89), or MMG MPF (*p* = 0.52). Polynomial regression analyses demonstrated substantial inter-individual variability. Inferences made regarding the patterns of neuromuscular responses to fatiguing and bilateral muscle actions should be considered on a subject-by-subject basis.

## 1. Introduction

Neuromuscular parameters from invasive [1] and non-invasive [2,3] assessments of muscle function have been used in laboratory and clinical settings to examine factors relating to physical performance [4] and muscular diseases [5]. Simultaneous measurements of surface electromyography (EMG) and mechanomyography (MMG) have been used to non-invasively examine numerous aspects of muscle function including muscle atrophy [6], sarcopenia [7], age-related changes in muscular performance [8,9], and fiber type characteristics [4,10] in healthy subjects. Furthermore, neuromuscular parameters have been used in a clinical setting to assess the influences of cerebral palsy [11], myotonic dystrophy [12], and Parkinson’s disease [13] on muscle function and to improve the control of human prosthetics [14,15]. Time and frequency domain parameters of the EMG and MMG signals provide unique information regarding the motor unit activation strategies that modulate force production. The amplitude and frequency content of the EMG signal reflect muscle activation [3] and action potential conduction velocity [16] respectively. The MMG signal is the mechanical counterpart of muscle activation as represented by the EMG signal. The amplitude and frequency of the MMG signal can reflect motor unit recruitment [2] and the global motor unit firing rate [17] of the activated unfused motor units, respectively.

Neuromuscular patterns of responses for EMG and MMG have been used to make inferences regarding the motor unit activation strategies that modulate force during fatiguing dynamic muscle actions [18,19,20,21]. For example, Smith et al. [18] reported increases in electromyographic (EMG) amplitude (AMP) and mechanomyographic (MMG) AMP, as well as decreases in EMG mean power frequency (MPF), and no change in MMG MPF from the vastus lateralis (VL) during 25 maximal, concentric, isokinetic leg extension muscle actions of the dominant limb at 120°·s^−1^. Camic et al. [19] reported decreases in EMG AMP, EMG MPF, MMG AMP, and MMG MPF from the VL during 30 maximal, concentric, isokinetic leg extension muscle actions from the dominant limb at 30°·s^−1^. Ebersole et al. [20] reported increases in EMG AMP and MMG AMP, as well as decreases in EMG MPF and MMG MPF from the VL during maximal, concentric, isokinetic leg extension muscle actions of the dominant limb at 60 and 300°·s^−1^. Perry-Rana et al. [21] demonstrated increases in EMG AMP, as well as decreases in MMG AMP from the VL during 50 maximal, concentric, isokinetic leg extension muscle actions of the dominant limb at 60, 180 and 300°·s^−1^. In general, these studies demonstrated that during unilateral, fatiguing, maximal, concentric, and isokinetic leg extension muscle actions, muscle activation (EMG AMP) exhibited quadratic or cubic increases [18,20,21], muscle fiber action potential conduction velocity (EMP MPF) [18,19,20], global motor unit firing rate (MMG MPF) [18,19] exhibited quadratic or cubic decreases, and motor unit recruitment (MMG AMP) exhibited conflicting patterns of responses. Thus, the assessment of the time and frequency domain parameters of EMG and MMG signals can provide unique information regarding motor unit activation strategies during fatiguing isokinetic muscle actions at various velocities.

Typically, the composite (data averaged across all subjects) patterns of neuromuscular responses have been used to make inferences regarding fatigue-induced changes in motor unit activation strategies [18,19,20,21,22,23]. Previous studies, however, have described great inter-individual differences in the patterns of responses from the VL for EMG AMP, EMG MPF, MMG AMP, and/or MMG MPF during ramp incremental [24,25] and step incremental [26,27,28,29,30,31,32] isometric muscle actions, concentric [33] and eccentric [34] isokinetic muscle actions, and incremental [35,36,37,38] and continuous [22] cycle ergometry that may be attributable to the inherent variability of EMG and MMG signals [24,39]. Furthermore, while previous studies [24,35] have demonstrated differences in the neuromuscular patterns of responses between synergistic muscles of ipsilateral limbs during unilateral muscle actions, there are limited data available regarding intra-individual differences for homologous muscles of contralateral limbs during fatiguing, bilateral muscle actions. Bilateral asymmetries in force production have been consistently demonstrated between dominant and non-dominant limbs, however, the cause of inter-limb asymmetries remains unclear [40]. Recent research has suggested that force asymmetries arise from the different specializations of the left and right hemispheres of the brain, which manifest as functional differences in the contralateral limbs [41]. It is unclear, however, whether inter-limb asymmetries in force production are reflected in neuromuscular parameters. During fatiguing, submaximal (20% of maximal voluntary contraction), bilateral, and isometric leg extension muscle actions, Matkowski et al. [42] reported no differences in the increase in EMG AMP from the VL or rectus femoris (RF) muscle of the left and right limbs. No previous studies, however, have examined the composite, inter-individual, or intra-individual variability of the fatigue-induced patterns of neuromuscular responses during bilateral, dynamic, muscle actions. Understanding the variability between these conditions will help to clarify whether inferences regarding the neuromuscular patterns of responses can be generalized between individuals, as well as between the limbs of an individual. Therefore, the purpose of this study was to compare the composite, inter-individual, and intra-individual differences in the patterns of responses for EMG AMP, EMG MPF, MMG AMP and MMG MPF during fatiguing, maximal, bilateral, and isokinetic leg extension muscle actions. Based on the results of previous investigations [24,33,42], we hypothesized that: 1) there will be no significant intra-individual differences in the patterns of neuromuscular responses between the VL muscles of the contralateral limbs; and 2) there would be inter-individual differences, as well as differences between individual and composite patterns of responses, for all neuromuscular parameters during the bilateral, fatiguing task.

## 2. Materials and Methods

### 2.1. Subjects

Thirteen healthy adult men (mean ± SD age = 21.7 ± 2.6 years; body mass = 79.8 ± 11.5 kg; height = 174.2 = 7 ± 12.7 cm) volunteered to participate in this study. The subjects were recreationally trained and participated in resistance and/or aerobic exercise at least 3 days a week [43]. All subjects were free from previous knee or ankle injuries that would potentially hinder performance. The study was approved by the University Institutional Review Board for Human Sciences, and all subjects completed a health questionnaire and signed an informed consent document before testing.

### 2.2. Protocol

The first visit was an orientation session where the subjects were familiarized with the testing protocol. During the familiarization, the subjects performed submaximal and maximal bilateral isometric and isokinetic leg extensions at 180°·s^−1^. For the test visits, the subjects warmed up by performing 5 submaximal isokinetic leg extensions at 180°·s^−1^ on a calibrated Cybex II dynamometer (Cybex, Division of Lumex, Inc., Ronkonkoma, NY, USA). The subjects then performed two bilateral maximal, voluntary isometric contractions (MVICs) for 6 s at a knee joint angle of 135° (full extension corresponds to 180°). This was followed by consecutive maximal, bilateral isokinetic leg extensions at 180°·s^−1^ until the torque values dropped to 50% of peak torque for two consecutive repetitions.

### 2.3. Electromyographic, Mechanomyographic, and Force Signal Acquisition

**Figure 1:**
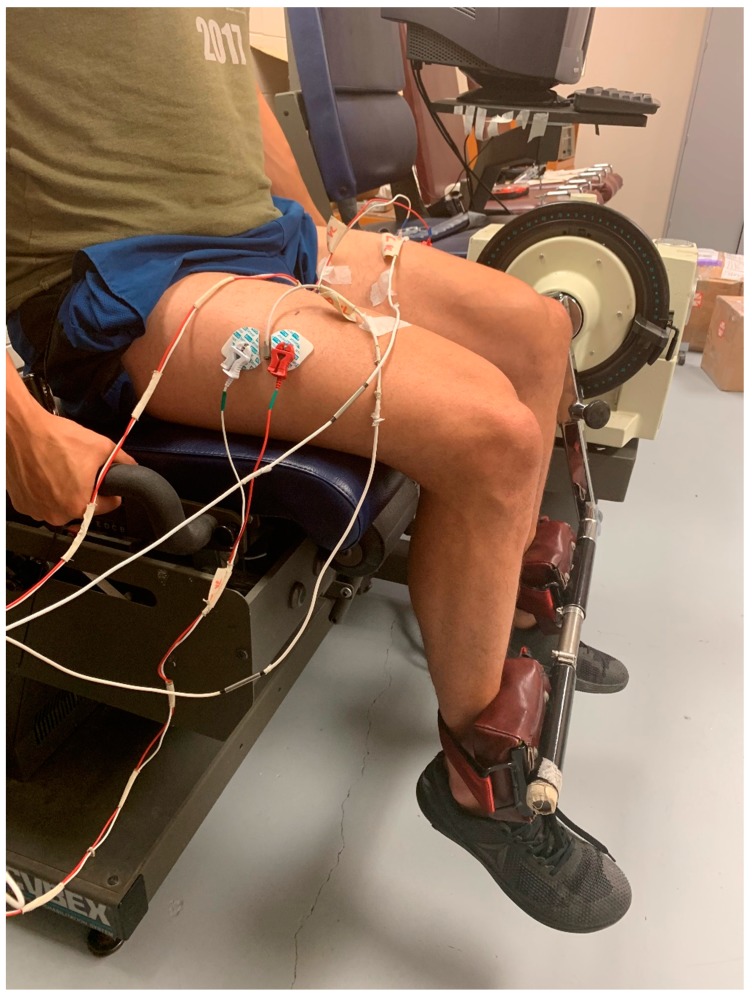
Image of the electrode and accelerometer arrangements for electromyographic (EMG) and mechanomyographic (MMG) signal acquisition for both vastus lateralis (VL) muscles during the fatiguing task.

During the test visit, bipolar (30-mm center-to-center) surface EMG electrode (circular 4-mm diameter silver/silver chloride; Biopac Systems, Inc, Santa Barbara, CA, USA) arrangements were placed on the VL muscles of both limbs according to the Surface Electromyography for the Non-Invasive Assessment of Muscles project (SENIAM) recommendations [44]. The electrodes were placed 66% of the distance between the anterior superior iliac spine and the lateral border of the patella and were oriented at a 20° angle to align with the angle of pennation of the VL muscle fibers (Figure 1) [45]. A reference electrode was placed over the anterior superior iliac spine. Prior to electrode placement, the skin was shaved, carefully abraded, and cleaned with alcohol. The MMG signals for both VL muscles were detected using miniature accelerometers (Entras EGAS FT 10, bandwidth 0–200 Hz, dimensions 1.0 × 1.0 × 0.5 cm, mass 1.0 g, sensitivity 668.1 mV·g^−1^ for the right VL, 655.1 mV·g^−1^ for the left VL) placed between the bipolar EMG arrangements of both VL muscles using double-sided adhesive tape (Figure 1).

The raw EMG and MMG signals were digitized at 2000 Hz with a 12-bit analog-to-digital converter (Model MP150; Biopac Systems, Inc.) and stored on a personal computer (G5 15 Dell Inc., Round Rock, TX, USA) for analyses. The EMG signals were amplified (gain: ×1000) using differential amplifiers (EMG2-R Bionomadix, Biopac Systems, Inc. Goleta, CA, USA; bandwidth—10–500 Hz). The EMG and MMG signals were digitally bandpass filtered (fourth-order Butterworth) at 10–500 Hz and 5–100 Hz, respectively. Signal processing was performed using custom programs written with the LabVIEW programming software (version 18.0f2, National Instruments, Austin, TX, USA). The EMG (µV root mean square, µVrms) and MMG (m·s^−2^) AMP and MPF (Hz) values for each leg extension muscle action were calculated for a period of time that corresponded to the middle 30° range of motion from approximately 120° to 150° of the leg extension, to avoid the acceleration and deceleration phases of the isokinetic muscle actions [46]. This corresponded to a signal epoch of 0.17 s used to calculate the AMP and MPF values of the EMG and MMG signals. The EMG and MMG, AMP and MPF values for the MVIC were calculated from the middle 2 s of the 6 s trial. The corresponding values from the MVIC with the highest torque output were used to normalize the EMG and MMG parameters for each repetition during the bilateral leg extensions. The repetitions were normalized to each 5% of the total number of repetitions completed. Torque values were normalized to the value at 10% of the total repetitions completed.

### 2.4. Statistical Analysis

Data analyses began at 10% of total repetitions completed to eliminate initial submaximal repetitions [47]. Four, 2 (Limb (right and left VL)) × 19 (Time (10–100% of the total repetitions at 5% intervals)) repeated measures ANOVAs were used to examine mean differences for each normalized neuromuscular parameter (EMG AMP, EMG MPF, MMG AMP, MMG MPF). A 1 × 19 repeated measures ANOVA was used to examine fatigue-related changes in normalized torque production. Post-hoc Student–Newman–Keuls [48] was used to identify if the normalized neuromuscular and torque values changed from the values at 10% of the total repetitions.

Separate polynomial regression analyses (linear and quadratic) were used to define the individual and composite normalized EMG AMP, EMG MPF, MMG AMP, MMG MPF for both limbs and the composite normalized torque values versus total repetitions relationships during the fatiguing protocol. All statistical analyses were performed using IBM SPSS v. 25 (Armonk, NY, USA). An alpha of *p* ≤ 0.05 was considered statistically significant for all comparisons.

## 3. Results

### 3.1. Torque Response

**Figure 2:**
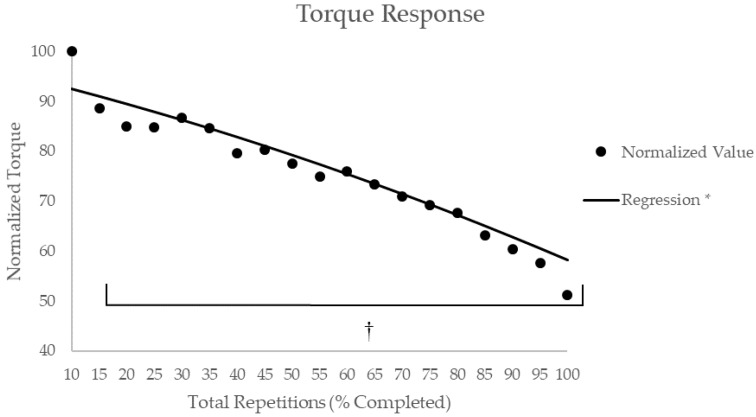
Time course of changes for the composite torque measures during maximal bilateral leg extensions. * Indicates there was a significant relationship (R = 0.95, *p* < 0.05) between normalized torque values and total repetitions as determined by polynomial regression. † Indicates significantly (*p* < 0.05) lower torque from the initial 10% of total repetitions completed based on post-hoc Student–Newman–Keuls mean comparisons.

The subjects performed a total of 56 ± 17 leg extension repetitions with the average peak torque of 326.5 ± 49.2 N·m. There was a significant effect for time (*p* < 0.01, η^2^_p_ = 0.78) for average peak torque, with post-hoc Student–Newman–Keuls analyses indicating that torque was significantly less for all values after the initial 10% of the total repetitions (Figure 2). Polynomial regression exhibited a significant, negative quadratic (R = 0.95) relationship for torque across total repetitions (Figure 2).

### 3.2. EMG Response

**Figure 3:**
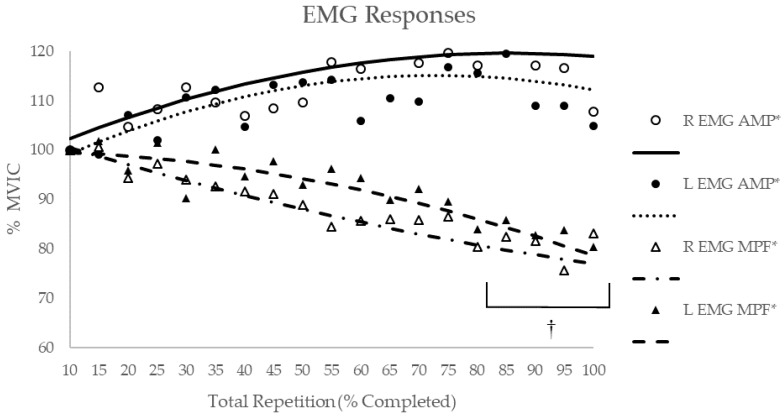
Time course of changes for EMG measures during maximal bilateral leg extensions for both limbs (R = right and L = left). * Indicates there was a significant (*p* < 0.05) relationship between normalized EMG values and the total repetitions as determined by polynomial regression. † Indicates significantly (*p* < 0.05) lower EMG mean power frequency (MPF) collapsed across limbs than the initial 10% of total repetitions completed based on post-hoc Student–Newman–Keuls mean comparisons.

The 2 (Limb (right and left VL)) × 19 (Time (10–100% of the total repetitions at 5% intervals)) repeated measures ANOVA for EMG AMP indicated no significant interaction (*p* = 0.91, η^2^_p_ = 0.05) or main effects for limb (*p* = 0.44, η^2^_p_ = 0.05) or time (*p* = 0.06, η^2^_p_ = 0.12). For EMG MPF, there was no significant interaction (*p* = 0.61, η^2^_p_ = 0.07) or main effect for the limb (*p* = 0.33, η^2^_p_ = 0.08), but there was a significant main effect for time (*p* < 0.01, η^2^_p_ = 0.32). Post-hoc Student–Newman–Keuls analyses indicated that EMG MPF at 80%, 85%, 90%, 95%, and 100% of the total repetitions was significantly less than the initial value at 10% of the total repetitions (Figure 3).

Polynomial regression analyses for the individual and composite EMG AMP responses (Table 1) for the right limb exhibited positive, quadratic relationships (R = 0.69 to 0.75) for 2 of the 13 subjects; a positive, linear relationship (r = 0.55) for one subject; no significant relationships for 10 subjects; and a positive, quadratic relationship (R = 0.75) for the composite data. For the left limb, 3 of the 13 subjects exhibited positive, quadratic relationships (R = 0.56 to 0.81); 10 subjects exhibited no significant relationships; and the composite data exhibited a positive, quadratic relationship (R = 0.79).

Polynomial regression analyses for the individual and composite EMG MPF responses (Table 1) for the right limb exhibited negative, quadratic relationships (R = −0.95 to −0.72) for 8 of the 13 subjects; a positive, quadratic relationship (R = 0.87) for one subject; no significant relationships for 4 subjects; and a negative, quadratic relationship (R = −0.95) for the composite data. For the left limb, 9 of the 13 subjects exhibited negative, quadratic relationships (R = −0.75 to −0.56); one subject exhibited a negative, linear relationship (r = −0.53); one subject exhibited a positive, quadratic relationship (R = 0.58); 2 subjects exhibited no significant relationships; and the composite data exhibited a negative, quadratic relationship (R = −0.91).

### 3.3. MMG Response

**Figure 4:**
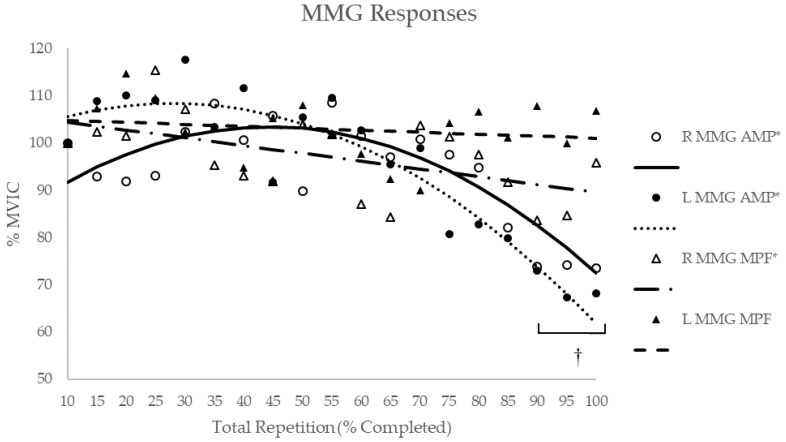
The time course of changes for MMG measures during maximal bilateral leg extensions for both limbs (R = right and L = left). * Indicates there was a significant (*p* < 0.05) relationship between normalized MMG values and repetitions as determined by polynomial regression. † Indicates significantly lower (p < 0.05) for MMG amplitude (AMP) collapsed between limbs from 30% of total repetitions completed, based on post-hoc Student–Newman–Keuls mean comparisons.

The 2 (Limb (right and left VL)) × 19 (Time (10–100% of the total repetitions at 5% intervals)) repeated measures ANOVAs for MMG AMP indicated no significant interaction (*p* = 0.34, η^2^_p_ = 0.09) or main effect for the limb (*p* = 0.89, η^2^_p_ = 0.00), but a significant main effect for time (*p* < 0.01, η^2^_p_ = 0.22). Post-hoc Student–Newman–Keuls indicated that MMG AMP at 90%, 95%, and 100% of total repetitions was significantly less than the peak value at 30% of total repetitions (Figure 4). For MMG MPF, there was no significant interaction (*p* = 0.93, η^2^_p_ = 0.05) or main effects for the limb (*p* = 0.52, η^2^_p_ = 0.04) or time (*p* = 0.62, η^2^_p_ = 0.07).

Polynomial regression analyses for the individual and composite MMG AMP responses (Table 2) for the right limb exhibited positive, quadratic relationships (R = 0.62 and 0.79) for 2 of the 13 subjects; a negative, quadratic relationship (R = −0.80 to −0.56) for 3 subjects; no significant relationships for 8 subjects; and a negative, quadratic relationship (R = −0.86) for the composite data. For the left limb, 5 of the 13 subjects exhibited negative, quadratic relationships (R = −0.87 to −0.56); one subject exhibited a positive quadratic relationship (R = 0.64); two subjects exhibited a negative, linear relationship (r = −0.496 and −0.469); 5 subjects exhibited no significant relationships; and the composite data exhibited a negative, quadratic relationship (R = −0.93).

Polynomial regression analyses for the individual and composite MMG MPF responses (Table 2) for the left limb exhibited negative, quadratic relationships (R = −0.89 to −0.60) for 3 of the 13 subjects; a negative, linear relationship (r = 0.51) for one subject; a positive, linear relationship (r = 0.48) for one subject; no significant relationships for 8 subjects; and a negative, linear relationship (r = −0.55) for the composite data. For the left limb, 1 of the 13 subjects exhibited a negative, quadratic relationship (R = −0.65); one subject exhibited a positive, quadratic relationship (r = 0.78); and 11 subjects and the composite data exhibited no significant relationships.

## 4. Discussion

The purpose of the present study was to compare the composite, inter-individual, and intra-individual differences in patterns of neuromuscular responses during dynamic muscle actions. The results indicated that there were no differences between limbs for any of the mean neuromuscular (EMG AMP, EMG MPF, MMG AMP, or MMG MPF) responses during the fatiguing, maximal, bilateral, or isokinetic leg extension muscle actions. This study is the first to compare the MMG, time and frequency domain responses in both limbs during fatiguing, bilateral muscle actions. However, previous studies [42,49,50,51] have examined EMG AMP and EMG MPF responses to bilateral muscle actions using various study designs. For example, Matkowski et al. [49] reported no differences between limbs for normalized EMG AMP (normalized to an electrically evoked M-wave during an MVIC) from the RF, vastus medialis (VM), and VL during bilateral leg extension MVICs. Matkowski et al. [42] also reported no difference between limbs for normalized EMG AMP from the RF and VL during sustained, submaximal, bilateral, or isometric muscle actions at 20% of MVIC. Oda and Moritani [50] found no differences between limbs for EMG AMP and EMG MPF from the biceps brachii following 64 s of sustained, bilateral forearm flexion MVICs. In addition, Post et al. [51] reported no differences between limbs for EMP AMP from the first dorsal interosseous muscles during bilateral MVICs. Thus, the results of the present study supported previous studies [42,49,50,51] that have reported no differences between homologous muscles of contralateral limbs for EMG AMP and/or EMG MPF during bilateral isometric muscle actions, and extended these findings to include MMG AMP and MMG MPF responses during isokinetic muscle actions.

The lack of difference between any of the neuromuscular parameters exhibited in the present study may be due to a common efferent input to the active skeletal muscle [52]. During corticomuscular activation, continuous interhemispheric communication between the cortices has been shown to elicit a “bilateral coupling” of the efferent signal to homologous muscles and result in similar neuromuscular activation [53,54]. During fatiguing bilateral tasks, increased intermuscular cross-correlation and coherence [55,56] suggests the bilateral coupling of the efferent signal—similar to a common drive described by De Luca et al. [57,58]—could be a mechanism to mitigate task failure. The results from the present study demonstrated similar neuromuscular patterns of responses in homologous muscles for the composite results during the fatiguing task, which was consistent with a bilateral coupling of the efferent signal to active skeletal muscle. Interlimb differences in the neuromuscular patterns of responses for individual subjects, however, may suggest the presence of intra-individual variation in efferent signaling during fatiguing, isokinetic, bilateral muscle actions.

In the present study, the composite data for EMG AMP and EMG MPF of both VL muscles demonstrated quadratic increase and quadratic decreases, respectively. Increases in EMG AMP during fatiguing muscle actions have been attributed to increases in muscle activation [59]. Decreases in EMG MPF have been attributed to the reductions in conduction velocity of the myoelectric signal [60], caused by a build-up of metabolic by-products such as extracellular potassium [61]. The composite results of this study indicated that throughout the fatiguing task, the decline in force was associated with increased muscle activation and the build-up of metabolic by-products that were similar in both limbs. The composite results for the MMG AMP of both VL muscles indicated quadratic decreases over the time course of the fatiguing task. Decreases in MMG AMP have been attributed to decreases in muscular compliance caused by increased muscle thickness, fluid content, and intramuscular pressure [62,63]. The composite data suggested the development of fatigue may have reduced muscle compliance, which restricted the lateral oscillation of the active muscle fibers and resulted in a decrease in MMG AMP. The composite results for the MMG MPF for the right leg indicated a linear decrease over the time course of the fatiguing task, while the left leg demonstrated no significant change over time. The MMG MPF qualitatively reflects the global firing rate of the active motor units [17,64]. The composite results indicated that the MMG MPF differed between the right and left leg during the fatiguing task.

The results of the composite regression analyses demonstrated the same fatigue-induced patterns of responses in both limbs for EMG AMP, EMG MPF, and MMG AMP. For MMG MPF, the right limb exhibited a negative, linear pattern of response, while the left limb exhibited no significant pattern of response. There were, however, inter-individual differences for the patterns of neuromuscular responses between limbs (Table 1 and Table 2). Specifically, 9 of the 13 subjects had the same fatigue-related patterns of response for EMG AMP between limbs; 8 of the 13 subjects had the same fatigue-related patterns of response for EMG MPF between limbs; and 6 of the 13 subjects had the same fatigue-related patterns of response for MMG AMP between limbs. Previous studies have demonstrated the importance of subject-by-subject analyses of EMG [39] and MMG [24], due to the inherent variability of the EMG and MMG signal during isometric contractions. The continuous change in the range of motion during isokinetic muscle actions further adds to the variability of the surface EMG signal [65,66] and likely the MMG signal. Cochrane-Snyman et al. [22] similarly demonstrated inter-individual variability for both EMG and MMG patterns of responses during fatiguing cycle ergometry. Thus, the results from the current study and future studies assessing fatiguing, isokinetic muscle actions should be interpreted on a subject-by-subject basis in addition to the composite analyses.

In the present study, the individual subjects’ patterns of neuromuscular responses differed from the patterns of responses exhibited by the composite results (Table 1 and Table 2). The composite EMG AMP data of both VL muscles demonstrated quadratic increases across repetitions, while MMG AMP and EMG MPF decreased quadratically. There was also a significant linear decrease across repetitions for the composite MMG MPF response of the right VL. For the 26 neuromuscular patterns of responses for both VL muscles (13 subjects × 2 VL muscles), approximately 23% of the individual responses resulted in significant increases for the EMG AMP versus total repetitions relationships. For MMG AMP, 50% of the individual responses had a significant MMG AMP versus total repetitions relationship; of those, approximately 80% had a negative relationship, with the remaining 20% having a positive relationship. For MMG MPF of the right VL muscle, approximately 39% of the individual responses exhibited a significant relationship during the fatiguing task; of those, approximately 80% of the subjects had a negative relationship, while 20% had a positive relationship. For EMG MPF, the composite results indicated a negative quadratic relationship and 77% of the individual responses had a significant relationship. Of those individuals, 90% had a negative relationship, while the remaining 10% had a positive relationship versus repetitions. These findings indicated that the significant fatigue-induced composite increases for EMG AMP of both VL muscles, decreases for MMG AMP of both VL muscles and decreases for MMG MPF of the right VL muscle were generally reflective of a minority of subjects (n = 2 for right EMG AMP, n = 3 for left EMG AMP, n = 3 for right MMG AMP, n = 5 for left MMG AMP, and n = 1 for right MMG MPF), while the majority of the patterns of neuromuscular responses demonstrated no significant relationships across total repetitions (Table 1 and Table 2). The individual EMG MPF patterns of response, however, were closely related to the composite pattern (n = 8 for the right VL and n = 10 for the left VL). Thus, interpreting fatigue-related changes in motor unit activation strategies from the composite patterns of EMG AMP, MMG AMP, and MMG MPF responses would not be consistent with the individual responses for most subjects. The EMG MPF patterns of responses were the most consistent between individual subjects, as well as between the individual and composite responses. The discrepancy between the neuromuscular patterns of responses between subjects in the present study may be indicative of interindividual variations in fatigue-related changes in motor unit activation strategies.

## 5. Conclusions

In conclusion, the results of this study demonstrated no significant differences in neuromuscular responses between limbs during fatiguing, maximal, bilateral leg extensions. There were, however, differences between subjects for both limbs and differences between the individual and the composite results for all of the neuromuscular parameters. In general, these findings demonstrated the importance of considering both the individual and composite results when attempting to interpret motor unit activation strategies. While both individual and composite results indicated fatigue-induced declines in EMG MPF, inter-individual variations for EMG AMP, MMG AMP, and MMG MPF suggested that the observed neuromuscular patterns of responses of the composite results may not be representative of the whole population under investigation. Future studies are warranted to further explore intra-individual neuromuscular control strategies during bilateral muscle actions and to determine the relationship between individual and composite interpretations of motor unit control strategies during maximal, dynamic, fatiguing tasks.

## Figures and Tables

**Table 1:** Polynomial regression model, correlation, and p-values for normalized EMG parameters for both limbs (R = right and L = left) during maximal, bilateral leg extensions to fatigue.

Subjects	R EMG AMP			L EMG AMP			R EMG MPF			L EMG MPF		
	Model	Correlation	*p*-Value	Model	Correlation	*p*-Value	Model	Correlation	*p*-Value	Model	Correlation	*p*-Value
1	–	–	–	–	–	–	Quadratic	−0.733	0.002	Quadratic	−0.563	0.047
2	Quadratic	0.685	0.006	Quadratic	0.831	<0.001	Quadratic	−0.733	0.002	Quadratic	−0.721	0.003
3	–	–	–	–	–	–	–	–	–	Quadratic	0.579	0.039
4	–	–	–	–	–	–	–	–	–	–	–	–
5	–	–	–	Quadratic	0.590	0.033	Quadratic	−0.949	<0.001	Quadratic	−0.749	0.001
6	–	–	–	–	–	–	Quadratic	0.870	<0.001	Quadratic	−0.585	0.035
7	Quadratic	0.749	0.001	–	–	–	Quadratic	−0.805	<0.001	Linear	−0.525	0.021
8	–	–	–	–	–	–	–	–	–	Quadratic	−0.619	0.021
9	–	–	–	Quadratic	0.562	0.048	Quadratic	−0.700	0.005	Quadratic	−0.739	0.002
10	–	–	–	–	–	–	–	–	–	Quadratic	−0.685	0.006
11	–	–	–	–	–	–	Quadratic	−0.715	0.003	Quadratic	−0.633	0.017
12	Linear	0.549	0.015	–	–	–	Quadratic	−0.778	0.001	Quadratic	−0.718	0.003
13	–	–	–	–	–	–	Quadratic	−0.767	0.001	–	–	–
Composite	Quadratic	0.749	0.001	Quadratic	0.793	0.002	Quadratic	−0.953	<0.001	Quadratic	−0.913	<0.001

**Table 2:** Polynomial regression model, correlation, and p-values for normalized MMG parameters for both limbs (R = right and L = left) during maximal, bilateral leg extensions to fatigue.

Subjects	R MMG AMP			L MMG AMP			R MMG MPF			L MMG MPF		
	Model	Correlation	*p*-Value	Model	Correlation	*p*-Value	Model	Correlation	*p*-Value	model	Correlation	*p*-Value
1	–	–	–	Linear	−0.498	0.030	Quadratic	−0.603	0.020	–	–	–
2	–	–	–	–	–	–	–	–	–	Quadratic	−0.658	0.011
3	–	–	–	–	–	–	–	–	–	–	–	–
4	Quadratic	−0.769	0.001	Quadratic	−0.679	0.001	–	–	–	–	–	–
5	–	–	–	–	–	–	Linear	−0.506	0.027	–	–	–
6	Quadratic	−0.804	<0.001	Quadratic	−0.759	0.001	Linear	0.480	0.037	Quadratic	0.779	0.001
7	–	–	–	Quadratic	−0.569	0.044	–	–	–	–	–	–
8	–	–	–	Quadratic	−0.867	<0.001	–	–	–	–	–	–
9	–	–	–	Quadratic	−0.651	0.012	–	–	–	–	–	–
10	Quadratic	0.615	0.022	Quadratic	0.637	0.015	Quadratic	−0.792	<0.001	–	–	–
11	–	–	–	Linear	−0.469	0.043	–	–	–	–	–	–
12	Quadratic	0.789	<0.001	–	–	–	Quadratic	−0.890	<0.001	–	–	–
13	Quadratic	−0.564	0.047	–	–	–	–	–	–	–	–	–
Composite	Quadratic	−0.864	<0.001	Quadratic	−0.933	<0.001	Linear	−0.547	0.015	–	–	–

## References

[1] Sperandeo M., Trovato F.M., Melillo N., Dimitri L., Musumeci G., Guglielmi G. (2017). The role of ultrasound-guided fine needle aspiration biopsy in musculoskeletal diseases. Eur. J. Radiol..

[2] Orizio C. (1993). Muscle sound: Bases for the introduction of a mechanomyographic signal in muscle studies. Crit. Rev. Biomed. Eng..

[3] De Luca C.J. (1997). The Use of Surface Electromyography in Biomechanics. J. Appl. Biomech..

[4] Hamada T., Sale D.G., MacDougall J.D., Tarnopolsky M.A. (2000). Postactivation potentiation, fiber type, and twitch contraction time in human knee extensor muscles. J. Appl. Physiol..

[5] Barry D.T., Gordon K.E., Hinton G.G. (1990). Acoustic and surface EMG diagnosis of pediatric muscle disease. Muscle Nerve.

[6] Marchetti M., Salleo A., Figura F., Del Gaudio V., Nelson R.C., Morehouse C.A. (1974). Electromyographic and phonomyographic patterns in muscle atrophy in man. Biomechanics IV: Proceedings of the Fourth International Seminar on Biomechanics.

[7] Tian S.-L., Liu Y., Li L., Fu W.-J., Peng C.-H. (2010). Mechanomyography is more sensitive than EMG in detecting age-related sarcopenia. J. Biomech..

[8] Esposito F., Malgrati D., Veicsteinas A., Orizio C. (1996). Time and frequency domain analysis of electromyogram and sound myogram in the elderly. Eur. J. Appl. Physiol..

[9] Musumeci G., Castrogiovanni P., Coleman R., Szychlinska M.A., Salvatorelli L., Parenti R., Magro G., Imbesi R. (2015). Somitogenesis: From somite to skeletal muscle. Acta Histochem..

[10] Marchetti M., Felici F., Bernardi M., Minasi P., Di Filippo L. (1992). Can evoked phonomyography be used to recognize fast and slow muscle in man?. Int. J. Sports Med..

[11] Akataki K., Mita K., Itoh K., Suzuki N., Watakabe M. (1996). Acoustic and electrical activities during voluntary isometric contraction of biceps brachii muscles in patients with spastic cerebral palsy. Muscle Nerve.

[12] Orizio C., Esposito F., Paganotti I., Marino L., Rossi B., Veicsteinas A. (1997). Electrically-elicited surface mechanomyogram in myotonic dystrophy. Ital. J. Neurol. Sci..

[13] Marusiak J., Jaskólska A., Kisiel-Sajewicz K., Yue G.H., Jaskólski A. (2009). EMG and MMG activities of agonist and antagonist muscles in Parkinson’s disease patients during absolute submaximal load holding. J. Electromyogr. Kinesiol..

[14] De la Rosa R., Alonso A., Carrera A., Durán R., Fernández P. (2010). Man-machine interface system for neuromuscular training and evaluation based on EMG and MMG signals. Sensors.

[15] Silva J., Heim W., Chau T. (2004). MMG-based classification of muscle activity for prosthesis control. Conf. Proc. Annu. Int. Conf. IEEE Eng. Med. Biol. Soc..

[16] De Luca C.J. (1984). Myoelectrical manifestations of localized muscular fatigue in humans. Crit. Rev. Biomed. Eng..

[17] Beck T.W., Housh T.J., Johnson G.O., Cramer J.T., Weir J.P., Coburn J.W., Malek M.H. (2007). Does the frequency content of the surface mechanomyographic signal reflect motor unit firing rates? A brief review. J. Electromyogr. Kinesiol..

[18] Smith C., Housh T., Herda T., Zuniga J., Camic C., Bergstrom H., Smith D., Weir J., Hill E., Cochrane K. (2016). Time Course of Changes in Neuromuscular Parameters During Sustained Isometric Muscle Actions. J. Strength Cond. Res..

[19] Camic C.L., Housh T.J., Zuniga J.M., Russell Hendrix C., Bergstrom H.C., Traylor D.A., Schmidt R.J., Johnson G.O. (2013). Electromyographic and mechanomyographic responses across repeated maximal isometric and concentric muscle actions of the leg extensors. J. Electromyogr. Kinesiol..

[20] Ebersole K.T., O’Connor K.M., Wier A.P. (2006). Mechanomyographic and electromyographic responses to repeated concentric muscle actions of the quadriceps femoris. J. Electromyogr. Kinesiol..

[21] Perry-Rana S.R., Housh T.J., Johnson G.O., Bull A.J., Berning J.M., Cramer J.T. (2002). MMG and EMG responses during fatiguing isokinetic muscle contractions at different velocities. Muscle Nerve.

[22] Cochrane-Snyman K.C., Housh T.J., Smith C.M., Hill E.C., Jenkins N.D.M., Schmidt R.J., Johnson G.O. (2016). Inter-individual variability in the patterns of responses for electromyography and mechanomyography during cycle ergometry using an RPE-clamp model. Eur. J. Appl. Physiol..

[23] Beck T.W., Housh T.J., Johnson G.O., Weir J.P., Cramer J.T., Coburn J.W., Malek M.H. (2004). Mechanomyographic and electromyographic amplitude and frequency responses during fatiguing isokinetic muscle actions of the biceps brachii. Electromyogr. Clin. Neurophysiol..

[24] Ryan E.D., Cramer J.T., Housh T.J., Beck T.W., Herda T.J., Hartman M.J., Stout J.R. (2007). Inter-individual variability among the mechanomyographic and electromyographic amplitude and mean power frequency responses during isometric ramp muscle actions. Electromyogr. Clin. Neurophysiol..

[25] Ryan E.D., Beck T.W., Herda T.J., Hartman M.J., Stout J.R., Housh T.J., Cramer J.T. (2008). Mechanomyographic amplitude and mean power frequency responses during isometric ramp vs. step muscle actions. J. Neurosci. Methods.

[26] Beck T.W., Housh T.J., Cramer J.T., Malek M.H., Mielke M., Hendrix R. (2008). The effects of the innervation zone and interelectrode distance on the patterns of responses for electromyographic amplitude and mean power frequency versus isometric torque for the vastus lateralis muscle. Electromyogr. Clin. Neurophysiol..

[27] Beck T.W., Housh T.J., Cramer J.T., Malek M.H., Mielke M., Hendrix R., Weir J.P. (2007). A comparison of monopolar and bipolar recording techniques for examining the patterns of responses for electromyographic amplitude and mean power frequency versus isometric torque for the vastus lateralis muscle. J. Neurosci. Methods.

[28] Beck T.W., Housh T.J., Cramer J.T., Malek M.H., Mielke M., Hendrix R., Weir J.P. (2008). Electrode shift and normalization reduce the innervation zone’s influence on EMG. Med. Sci. Sports Exerc..

[29] Beck T.W., Housh T.J., Cramer J.T., Weir J.P. (2007). The effect of the estimated innervation zone on EMG amplitude and center frequency. Med. Sci. Sports Exerc..

[30] Beck T.W., Housh T.J., Cramer J.T., Weir J.P. (2008). The effects of electrode placement and innervation zone location on the electromyographic amplitude and mean power frequency versus isometric torque relationships for the vastus lateralis muscle. J. Electromyogr. Kinesiol. Off. J. Int. Soc. Electrophysiol. Kinesiol..

[31] Camic C., Housh T., Zuniga J.M., Hendrix R., Mielke M., Johnson G., Schmidt R. (2010). The influence of electrode orientation on the electromyographic amplitude and mean power frequency versus isometric torque relationships for the vastus lateralis. J. Exerc. Physiol. Online.

[32] Herda T.J., Housh T.J., Weir J.P., Ryan E.D., Costa P.B., Defreitas J.M., Walter A.A., Stout R.J., Beck T.W., Cramer J.T. (2009). The consistency of ordinary least-squares and generalized least-squares polynomial regression on characterizing the mechanomyographic amplitude versus torque relationship. Physiol. Meas..

[33] Beck T.W., Housh T.J., Mielke M., Cramer J.T., Weir J.P., Malek M.H., Johnson G.O. (2007). The influence of electrode placement over the innervation zone on electromyographic amplitude and mean power frequency versus isokinetic torque relationships. J. Neurosci. Methods.

[34] Camic C.L., Housh T.J., Zuniga J.M., Bergstrom H.C., Schmidt R.J., Johnson G.O. (2014). Mechanomyographic and electromyographic responses during fatiguing eccentric muscle actions of the leg extensors. J. Appl. Biomech..

[35] Malek M.H., Coburn J.W., Weir J.P., Beck T.W., Housh T.J. (2006). The effects of innervation zone on electromyographic amplitude and mean power frequency during incremental cycle ergometry. J. Neurosci. Methods.

[36] Malek M.H., Housh T.J., Coburn J.W., Weir J.P., Schmidt R.J., Beck T.W. (2006). The effects of interelectrode distance on electromyographic amplitude and mean power frequency during incremental cycle ergometry. J. Neurosci. Methods.

[37] Zuniga J.M., Housh T.J., Camic C.L., Hendrix C.R., Mielke M., Schmidt R.J., Johnson G.O. (2010). The effects of accelerometer placement on mechanomyographic amplitude and mean power frequency during cycle ergometry. J. Electromyogr. Kinesiol. Off. J. Int. Soc. Electrophysiol. Kinesiol..

[38] Zuniga J.M., Housh T.J., Hendrix C.R., Camic C.L., Mielke M., Schmidt R.J., Johnson G.O. (2009). The effects of electrode orientation on electromyographic amplitude and mean power frequency during cycle ergometry. J. Neurosci. Methods.

[39] Farina D., Merletti R., Enoka R.M. (2004). The extraction of neural strategies from the surface EMG. J. Appl. Physiol..

[40] Bishop C., Turner A., Read P. (2018). Effects of inter-limb asymmetries on physical and sports performance: A systematic review. J. Sports Sci..

[41] Yen S.-C., Olsavsky L.C., Cloonan C.M., Llanos A.R., Dwyer K.J., Nabian M., Farjadian A.B. (2018). An examination of lower limb asymmetry in ankle isometric force control. Hum. Mov. Sci..

[42] Matkowski B., Place N., Martin A., Lepers R. (2011). Neuromuscular fatigue differs following unilateral vs bilateral sustained submaximal contractions. Scand. J. Med. Sci. Sports.

[43] Riebe D., Ehrman J.K., Liguori G., Magal M., American College of Sports Medicine (2018). ACSM’s Guidelines for Exercise Testing and Prescription.

[44] Hermens H.J., Freriks B., Disselhorst-Klug C., Rau G. (2000). Development of recommendations for SEMG sensors and sensor placement procedures. J. Electromyogr. Kinesiol. Off. J. Int. Soc. Electrophysiol. Kinesiol..

[45] Abe T., Kumagai K., Brechue W.F. (2000). Fascicle length of leg muscles is greater in sprinters than distance runners. Med. Sci. Sports Exerc..

[46] Brown L.E., Whitehurst M., Gilbert R., Buchalter D.N. (1995). The effect of velocity and gender on load range during knee extension and flexion exercise on an isokinetic device. J. Orthop. Sports Phys. Ther..

[47] Brown L.E. (2000). Isokinetics in Human Performance.

[48] Keppel G., Wickens T. (2004). Design and Analysis.

[49] Matkowski B., Martin A., Lepers R. (2011). Comparison of maximal unilateral versus bilateral voluntary contraction force. Eur. J. Appl. Physiol..

[50] Oda S., Moritani T. (1995). Cross-correlation of bilateral differences in fatigue during sustained maximal voluntary contraction. Eur. J. Appl. Physiol..

[51] Post M., van Duinen H., Steens A., Renken R., Kuipers B., Maurits N., Zijdewind I. (2007). Reduced cortical activity during maximal bilateral contractions of the index finger. NeuroImage.

[52] Conway B.A., Halliday D.M., Farmer S.F., Shahani U., Maas P., Weir A.I., Rosenberg J.R. (1995). Synchronization between motor cortex and spinal motoneuronal pool during the performance of a maintained motor task in man. J. Physiol..

[53] Chang Y.-J., Chou C.-C., Chan H.-L., Hsu M.-J., Yeh M.-Y., Fang C.-Y., Chuang Y.-F., Wei S.-H., Lien H.-Y. (2012). Increases of quadriceps inter-muscular cross-correlation and coherence during exhausting stepping exercise. Sensors.

[54] Maudrich T., Kenville R., Lepsien J., Villringer A., Ragert P. (2018). Structural neural correlates of physiological mirror activity during isometric contractions of non-dominant hand muscles. Sci. Rep..

[55] Boonstra T.W., Daffertshofer A., van As E., van der Vlugt S., Beek P.J. (2007). Bilateral motor unit synchronization is functionally organized. Exp. Brain Res..

[56] Boonstra T.W., Daffertshofer A., van Ditshuizen J.C., van den Heuvel M.R.C., Hofman C., Willigenburg N.W., Beek P.J. (2008). Fatigue-related changes in motor-unit synchronization of quadriceps muscles within and across legs. J. Electromyogr. Kinesiol..

[57] De Luca C.J., Erim Z. (1994). Common drive of motor units in regulation of muscle force. Trends Neurosci..

[58] De Luca C.J., Erim Z. (2002). Common drive in motor units of a synergistic muscle pair. J. Neurophysiol..

[59] Basmajian J.V., De Luca C.J. (1985). Muscles Alive: Their Functions Revealed by Electromyography.

[60] Arendt-Nielsen L., Mills K.R. (1985). The relationship between mean power frequency of the EMG spectrum and muscle fibre conduction velocity. Electroencephalogr. Clin. Neurophysiol..

[61] Fortune E., Lowery M.M. (2009). Effect of extracellular potassium accumulation on muscle fiber conduction velocity: A simulation study. Ann. Biomed. Eng..

[62] Sjogaard G., Saltin B. (1982). Extra- and intracellular water spaces in muscles of man at rest and with dynamic exercise. Am. J. Physiol. Regul. Integr. Comp. Physiol..

[63] Jensen Z.R., Jørgensen K., Sjøgaard G. (1994). The effect of prolonged isometric contractions on muscle fluid balance. Eur. J. Appl. Physiol..

[64] Orizio C., Gobbo M., Diemont B., Esposito F., Veicsteinas A. (2003). The surface mechanomyogram as a tool to describe the influence of fatigue on biceps brachii motor unit activation strategy. Historical basis and novel evidence. Eur. J. Appl. Physiol..

[65] Earp J.E., Newton R.U., Cormie P., Blazevich A.J. (2013). Knee angle-specific EMG normalization: The use of polynomial based EMG-angle relationships. J. Electromyogr. Kinesiol. Off. J. Int. Soc. Electrophysiol. Kinesiol..

[66] Farina D. (2006). Interpretation of the surface electromyogram in dynamic contractions. Exerc. Sport Sci. Rev..

